# Synergistic interaction between wheat streak mosaic virus and Triticum mosaic virus modulates wheat transcriptome to favor disease severity

**DOI:** 10.3389/fpls.2024.1504482

**Published:** 2025-01-08

**Authors:** Haritha Nunna, Nathan A. Palmer, Gautam Sarath, Stephen N. Wegulo, Satyanarayana Tatineni

**Affiliations:** ^1^ Department of Plant Pathology, University of Nebraska-Lincoln, Lincoln, NE, United States; ^2^ United States Department of Agriculture-Agricultural Research Service (USDA-ARS), Wheat, Sorghum, and Forage Research Unit, Lincoln, NE, United States

**Keywords:** wheat, wheat streak mosaic virus, Triticum mosaic virus, synergistic interaction, disease synergism, transcriptome, transcription factors

## Abstract

Wheat streak mosaic virus (WSMV; *Tritimovirus tritici*) and Triticum mosaic virus (TriMV; *Poacevirus tritici*), the type members of the genera *Tritimovirus* and *Poacevirus*, respectively, in the family *Potyviridae*, are economically important wheat viruses in the Great Plains region of the USA. Co-infection of wheat by WSMV and TriMV results in disease synergism. Wheat transcriptome from singly (WSMV or TriMV) and doubly (WSMV+TriMV) infected upper uninoculated leaves were analyzed by RNA-Seq at 9, 12, and 21 days postinoculation. A total of 31,754 differentially expressed wheat genes were identified among all comparisons. Weighted gene co-expression network analysis resulted in 11 co-expression modules that broadly indicated gene expression profiles attributable to control, single, and double infections. Gene ontology, protein domain and KEGG (Kyoto Encyclopedia of Genes and Genomes) pathway enrichment analysis revealed that genes specifically related to photosynthesis, growth, stress, senescence, and defense were differentially enriched. Analyses of transcription factor families indicated that genes encoding MADS-Box and ARFs were strongly enriched in control plants, moderately repressed in TriMV-infected plants, and more strongly repressed in WSMV- and doubly-infected plants, whereas genes encoding WRKYs and NACs were more enriched in WSMV or doubly infected plants. Synergistic interactions between WSMV and TriMV drastically enhanced disease phenotype compared to individual virus infections. The progression of disease phenotype was correlated to transcriptomic changes, indicating the strong disruption to plant metabolism and likely channeling of energy and metabolites for viral replication. There also appeared to be a connection between viral replication and plastid health, with stronger downregulation of genes needed for chloroplast functions and integrity and increased synergism between TriMV and WSMV. This study provides an overview of transcriptomic changes distinctly influenced by TriMV and WSMV either singly or in combination and provides a good correlation between specific transcription factors and genes associated with metabolism to observed phenotypic changes in plant growth and disease synergism.

## 1 Introduction

Wheat (*Triticum aestivum* L.) is the second most cultivated cereal crop after maize, with a need to increase wheat production to meet the projected increase in population growth to 10 billion by 2050 (Godfray et al., 2010; Jones and Naidu, 2019). Apart from climatic changes, biotic stress caused by pathogens results in extensive yield losses, leading to an economic loss of about US$220 billion annually (Ristaino et al., 2021). Among viruses infecting wheat, wheat streak mosaic virus (WSMV), barley yellow dwarf virus, Triticum mosaic virus (TriMV), High Plains wheat mosaic virus, and wheat soil-borne mosaic virus are economically important in the Great Plains Region of the USA (Burrows et al., 2009).

WSMV (*Tritimovirus tritici*) and TriMV (*Poacevirus tritici*) are the type members of the genera *Tritimovirus* and *Poacevirus*, respectively, in the family *Potyviridae* (Stenger et al., 1998; Tatineni et al., 2009). WSMV is the most economically important wheat virus in the Great Plains region of the USA (Brakke, 1987; Singh et al., 2018), while TriMV was reported in 2008 and is widespread in the Great Plains region. TriMV is predominantly co-infected with WSMV in growers’ fields (Seifers et al., 2008; Byamukama et al., 2013). Both these viruses are transmitted by the wheat curl mite (*Aceria tosichella* Keifer) (Slykhuis, 1955; Seifers et al., 2009). The genomes of WSMV and TriMV are single-stranded positive-sense RNAs of 9.4 and 10.2 kb, respectively, which encode a large polyprotein that gets cleaved into at least 10 mature proteins by three virus-encoded proteinases, namely, P1, HC-Pro, and NIa-Pro (Stenger et al., 1998; Fellers et al., 2009; Tatineni et al., 2009). The HC-Pro and coat protein (CP) cistrons of WSMV and HC-Pro of TriMV have been reported as viral determinants of wheat curl mite transmission (Stenger et al., 2005; Tatineni and Hein, 2018; Tatineni et al., 2024). The CP and NIa-Pro cistrons of WSMV and TriMV are reported as elicitors of superinfection exclusion (Tatineni and French, 2016). The 6K1, NIa-VPg, NIa-Pro, and CP cistrons of WSMV have been identified as determinants of pathogenicity (Tatineni et al., 2023).

Insect vectors can carry and transmit more than one virus, leading to mixed viral infections in plants (Whitfield et al., 2015). Some mixed infections in plants result in disease synergism with exacerbated disease phenotype and increased titer of one or both interacting viruses (Syller, 2012; Mascia and Gallitelli, 2016; Moreno and López-Moya, 2020). Since WSMV and TriMV are transmitted by wheat curl mites, mixed infections are common in growers’ wheat fields (Byamukama et al., 2013). WSMV and TriMV synergistically interact in co-infected wheat with exacerbated disease phenotype and enhanced accumulation of both viruses compared to individual virus infections (Byamukama et al., 2012; Tatineni et al., 2010; Wong et al., 2025). Single and dual infections by TriMV and WSMV affected the biology of wheat plants (Tatineni et al., 2010, Tatineni et al., 2019; Soylu et al., 2024). Tatineni et al. (Tatineni et al., 2019) established an asymmetric interaction between TriMV and WSMV during the early stage of infection, depending on the order of infection of the viruses. Prior infection of wheat plants with TriMV enhanced the infectivity of WSMV, whereas prior infection by WSMV delayed the infection and accumulation of TriMV. Dual infections of wheat plants with WSMV and TriMV caused a differential accumulation of miRNAs, with significant upregulation of miRNAs 9670-3p, 397-5p, and 5384-3p and downregulation of miRNAs 319, 9773, and 9774. More specifically, miRNAs 397-5p, 398, and 9670-3p were associated with viral infections and predicted to target genes required for the defense and regulation of transcription (Soylu et al., 2024). Recently, Wong et al. (2025) reported that the synergistic interaction between WSMV and TriMV in co-infected wheat is not caused by the suppression of the host’s post-transcriptional gene silencing by the two viral RNA silencing suppressor proteins. Instead, the P1 and NIa-Pro proteins from both viruses work together to drive these synergistic interactions in wheat. These studies have provided important new findings on different aspects of the synergistic interactions between WSMV and TriMV.

RNA sequencing has been used to elucidate complex molecular mechanisms involved in virus-host interaction, such as understanding virus ecology in their natural ecosystem, identifying mutations, and global changes to plant transcriptomes upon virus infection (Han et al., 2015; Chen et al., 2016; Collum et al., 2016; Kamitani et al., 2016; Zhang et al., 2021; Dorostkar et al., 2022; Sharaf et al., 2023). Several other virus-host and virus-vector transcriptomic studies have been reported in wheat (Gupta et al., 2019; Xie et al., 2022; Soylu et al., 2024). However, an investigation of the transcriptome of wheat plants singly and doubly infected with WSMV and TriMV has yet to be reported. In this study, we analyzed global gene expression profiles in singly (WSMV or TriMV) and doubly (WSMV+TriMV) infected wheat plants.

## 2 Materials and methods

### 2.1 Inoculation of wheat seedlings with viruses

WSMV isolate Sydney 81 and TriMV isolate Nebraska were used in this study. Wheat leaves infected with *in vitro* transcripts of WSMV (Choi et al., 1999) or TriMV (Tatineni et al., 2015) at 14 days post-inoculation (dpi) were collected and stored at -80°C for future inoculations. The experimental design consisted of four treatments in a randomized block design (Mock, WSMV, TriMV, and WSMV+TriMV). Wheat cv. Tomahawk seedlings at the single-leaf stage were mechanically inoculated with crude sap of WSMV, TriMV, or both viruses at 1:20 (wt/v) in 20 mM sodium phosphate buffer, pH 7.0. Control plants were inoculated with 20 mM sodium phosphate buffer, pH 7.0. The virus-inoculated wheat plants were further grown in a greenhouse at 22 to 25°C with 16 h of natural or artificial light.

### 2.2 Tissue collection and RNA extraction

The upper noninoculated leaves from mock, WSMV, TriMV, and WSMV+TriMV-infected wheat plants were collected at 9, 12, and 21 dpi. Collected wheat leaves were flash-frozen in liquid N_2_ and stored at -80°C until needed. Three biological replicates were collected for each treatment and time point combination, resulting in a total of 36 samples for transcriptome analyses (4 treatments x 3 sampling dates x 3 replicates per treatment per sampling date). The frozen leaf tissue was cryogenically ground using liquid nitrogen in a mortar with a pestle. One hundred milligrams of powdered tissue was weighed into 1.5 mL microfuge tubes, and total RNA was extracted using the Total RNA extraction mini prep kit (Zymo Research, CA, USA). The extracted RNA samples were quantified and sent to Novogene Corporation Inc. (Sacramento, CA) for 2x150 PE Illumina sequencing. Raw sequencing reads have been deposited in the Sequence Read Archive under BioProject PRJNA1165236.

### 2.3 RNA seq analysis

Raw reads were trimmed using bbduk with the following parameters: k=13, ktrim=r, ftl=11, ftm=5, useshortkmers=t, mink=5, qtrim=t, trimq=10, and minlength=30. The trimmed reads were aligned to the wheat genome (Chinese Spring, v1.1; https://wheat-urgi.versailles.inra.fr) using STAR (Dobin et al., 2013) in two-pass mode, and unmapped reads were aligned to the WSMV (GI: 9630315) and TriMV (GI:239828769) genomes. The mapped reads were counted using FeatureCounts (Liao et al., 2014), and only reads mapping to annotated exon features were counted. Differential expression testing was performed using DESeq2 (Love et al., 2014), and a gene was regarded as differentially expressed when a contrast false discovery rate (FDR) corrected p-value was less than 0.05 and |log_2_FC| was greater than 1 for up- and below -1 for down-regulated genes. To visualize the differentially expressed genes (DEGs) common among treatments Venn diagrams were generated using a Ghent Venn diagram (https://bioinformatics.psb.ugent.be/webtools/Venn/). Weighted gene co-expression network analysis (Langfelder and Horvath, 2008) was used to generate coexpression networks with the following parameters: power=20, networkType=“signed”, TOMType=“signed”, and mergeCutHeight = 0.3. Domain and KEGG pathway (Kyoto Encyclopedia of Genes and Genomes) (Kanehisa and Goto, 2000) enrichment analysis was performed using the GeneOverlap package in R and the Interpro domain and KEGG orthology annotations included with the reference genome. Domains and KEGG pathways not found at least 10 times in the expressed gene set were excluded from the analysis. Gene ontology enrichment was performed using ShinyGO (Ge et al., 2020) with an FDR cut-off of 0.05.

## 3 Results

### 3.1 Phenotypic responses of wheat to single and double infections by WSMV and TriMV

**Figure 1:**
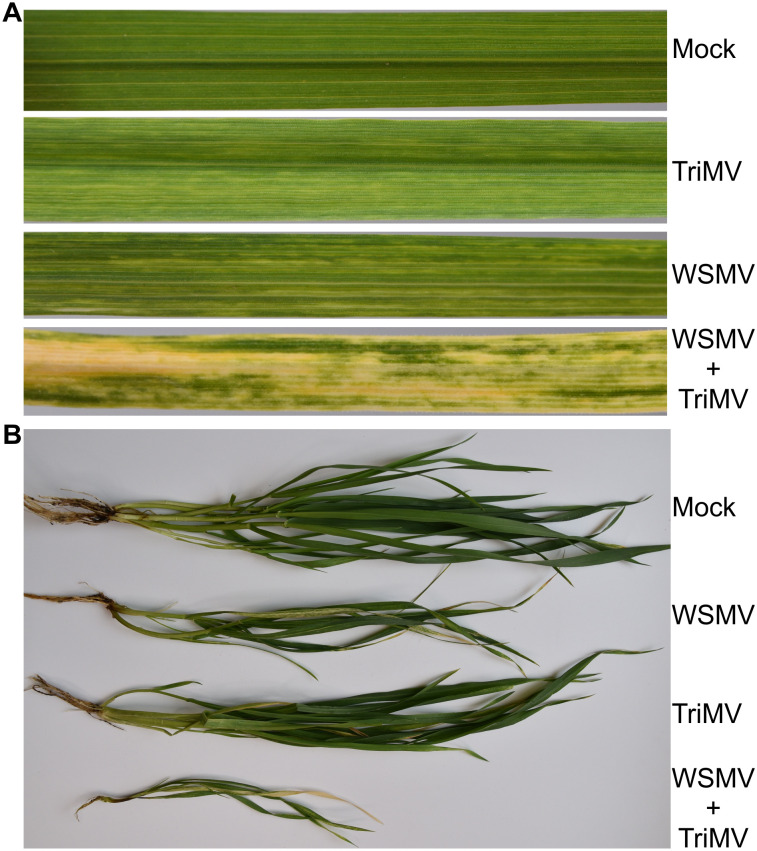
Symptom phenotype of wheat streak mosaic virus (WSMV), Triticum mosaic virus (TriMV), and Dual infection (WSMV+TriMV) in wheat cv. Tomahawk. **(A)** Wheat leaves showing symptoms of TriMV, WSMV, or both at 21 dpi. **(B)** Wheat plants showing the stunting symptoms elicited by WSMV, TriMV, or WSMV+TriMV at 21 dpi.

Phenotypic descriptions of wheat plants infected by WSMV, TriMV, or both at 9, 12, and 21 dpi are shown in **Table 1**. At 9 dpi, TriMV elicited mild mosaic and mottling symptoms, while WSMV produced chlorotic streaks in addition to mild mosaic and mottling symptoms (**Table 1**). In contrast, doubly infected plants showed moderate chlorotic streaks with moderate mosaic and mottling symptoms with no leaf yellowing. At 9 dpi, no virus combination induced noticeable stunting of plants. At 12 dpi, mosaic, mottling, chlorotic streaks, and stunting symptoms increased in all virus combinations, with symptom severity being greatest in doubly infected plants, followed by WSMV- and TriMV-infected plants in decreasing order of severity of symptoms. At 21 dpi, TriMV elicited mild stunting of plants with little or no leaf yellowing symptoms, while WSMV-infected plants developed mild leaf yellowing with moderate stunting of plants. The doubly infected plants showed significant leaf yellowing, mottling, and stunting of plants (**Figure 1**). Representative examples of leaves and whole plants from different treatments at 21 dpi are shown in **Figure 1**. Additionally, at 21 dpi, co-infection of wheat with TriMV and WSMV caused severe leaf deformation, and plants were severely stunted with shortened internodes (**Figure 1B**). At 28 dpi, many of the doubly infected plants were dead (data not shown).

**Table 1:** Symptom description of singly and doubly infected wheat by TriMV and WSMV at 9, 12, and 21 days postinoculation (dpi)^1^.

dpi	TriMV	WSMV	WSMV+ TriMV
9	Mild mosaic, mottling, and chlorotic streaks with no leaf yellowing symptoms and no stunting of plants	Mild mosaic and chlorotic streaks with no leaf yellowing symptoms and no stunting of plants	Moderate mosaic and mottling with chlorotic streaks; no leaf yellowing and no stunting of plants
12	Mild to moderate mosaic with mild chlorotic streaks; no leaf yellowing and no stunting of plants	Moderate chlorotic streaks and mosaic with no leaf yellowing symptoms; mild stunting of plants	Moderate to severe chlorotic streaks, mosaic, and mottling symptoms with mild leaf yellowing; mild stunting of plants
21	Moderate mosaic, mottling, and chlorotic streaks with no leaf yellowing symptoms; mild stunting of plants	Moderate chlorotic streaks and mosaic with mild leaf yellowing symptoms; moderate stunting of plants	Severe chlorotic streaks, mosaic, and mottling with severe leaf yellowing symptoms, leaf deformation, and shortened internodes resulted in severe stunting of plants

^1^Wheat cv. Tomahawk seedlings at the single-leaf stage were inoculated with crude sap extracted from infected wheat leaves at a 1:20 (w/v) dilution in 20 mM sodium phosphate buffer, pH 7.0. Note that dual infection of wheat by WSMV and TriMV elicited drastically more severe symptoms compared to individual virus infections.

### 3.2 Modulation of wheat transcriptome by single or double virus infection

**Figure 2:**
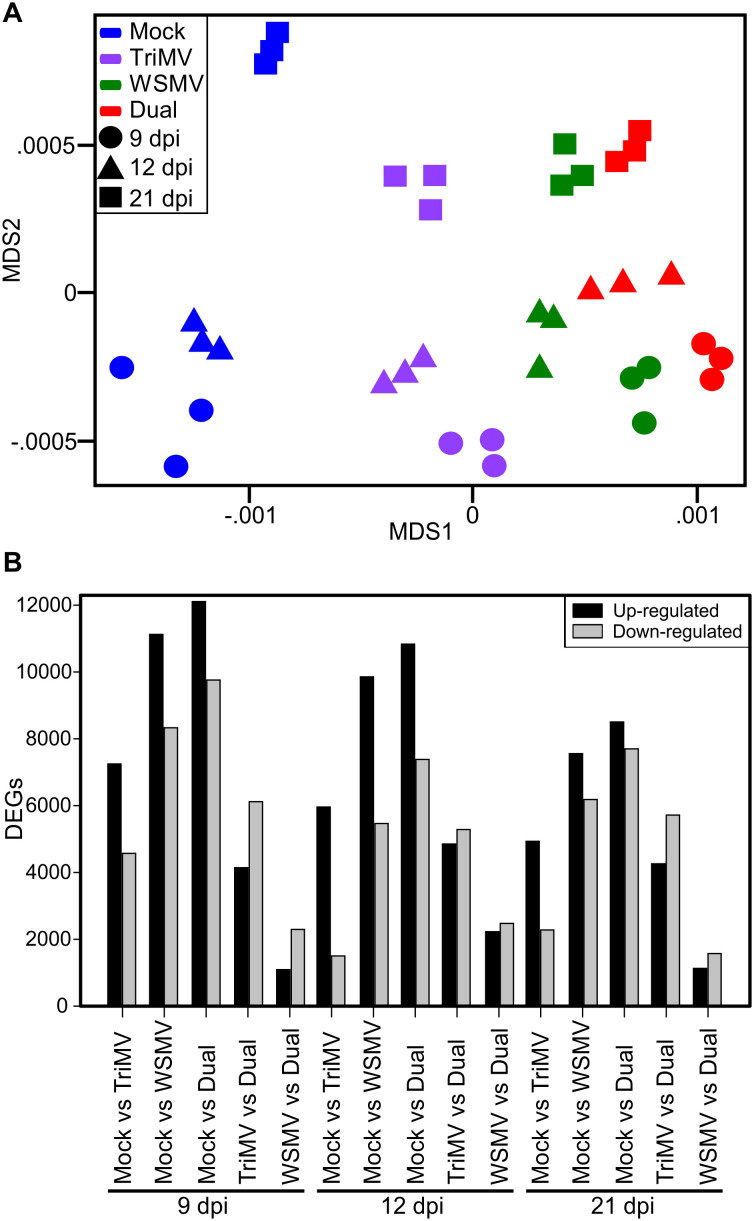
Overview of differentially expressed genes (DEGs) in wheat infected by wheat streak mosaic virus (WSMV), Triticum mosaic virus (TriMV), or both at 9, 12, and 21 days postinoculation (dpi). **(A)** Multidimensional scaling (MDS) plot showing the clustering of biological replicates where the X-axis separates the samples by treatment and the Y-axis separates the samples by days postinoculation. Each time point is depicted by shapes. 9 dpi=Circle, 12 dpi=Triangle, and 21 dpi=Square. Treatment is depicted by colors: Mock=Blue, TriMV=Violet, WSMV=Green, and double infection=Red. **(B)** Bar chart depicting the number of differentially expressed genes (DEGs) in wheat transcriptome in response to WSMV, TriMV, and dual virus infection relative to buffer inoculated plants at 9, 12, and 21 dpi.

RNA-Seq yielded an average of 54 million read-pairs per sample, which had an average mapping rate of 87% to the wheat (Chinese Spring, v1.1; https://wheat-urgi.versailles.inra.fr), WSMV (Stenger et al., 1998), and TriMV (Tatineni et al., 2009) genomes. Of the reads that mapped to the wheat genome, an average of 89.7% mapped to high-confidence annotated genes. A multidimensional scaling (MDS) plot of the experimental samples showed the clear clustering of the biological replicates within each treatment and sampling date, with the x-axis separating by treatment and the y-axis separating by time point (**Figure 2A**). The separation of samples by MDS indicated concurrence with the differential effects of single versus double infections. TriMV appeared to be less damaging as a single virus compared to WSMV, and dual viruses caused significantly greater effects on wheat plants (See **Table 1**; **Figure 1**). To identify treatment effects on gene expression, contrasts were tested between mock vs. WSMV, mock vs. TriMV, mock vs. double infection, WSMV vs. double infection, and TriMV vs. double infection for each time point. Genes were first classified as differentially expressed genes (DEGs) if their FDR corrected p-value was <0.05 and |log_2_FC|>1. Using these parameters, a total of 31,754 DEGs were detected across all treatments and time points. The number of genes upregulated (induced by infection) and downregulated (suppressed by infection) in each treatment are shown in **Figure 2B**. In general, double infections resulted in the highest number of DEGs at all three time points, followed by WSMV infection, with TriMV infection resulting in the least number of DEGs across all time points. There was also a decrease in total DEGs across time points, with 9 dpi having the most DEGs within each treatment and 21 dpi having the least DEGs within each treatment (**Figure 2B**).

### 3.3 Weighted gene co-expression network analysis (WGCNA)

**Figure 3:**
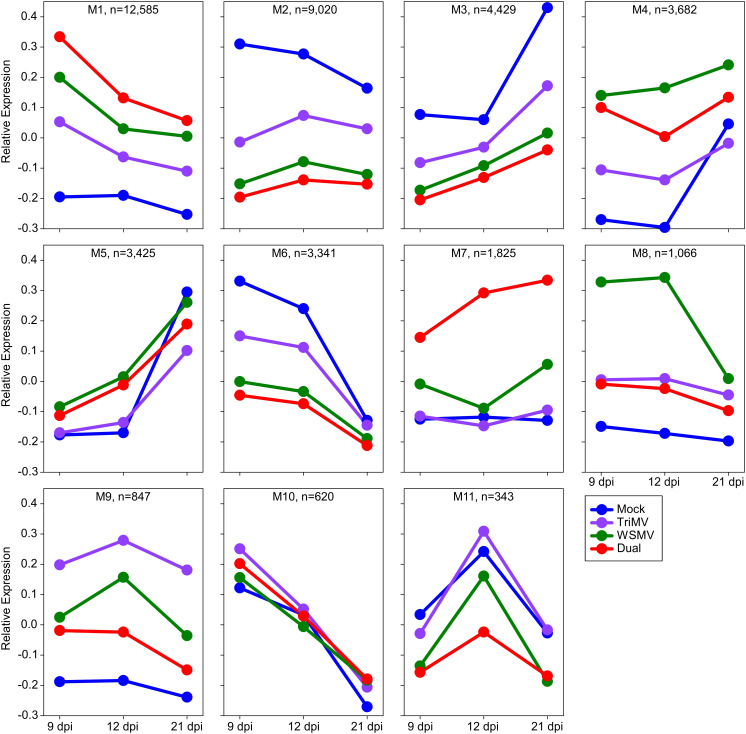
Weighted Gene Coexpression Network Analysis (WGCNA) of differentially expressed genes in wheat infected by wheat streak mosaic virus (WSMV), Triticum mosaic virus (TriMV), or both. M1 to M11 modules showed expression patterns of a set of genes expressed at 9, 12, and 21 days postinoculation (dpi). The dots represent the time points, and the colors represent the treatments. Blue=Mock, Violet=TriMV, Green=WSMV, and Red=Double infection.

WGCNA resulted in the detection of 11 co-expression modules among the DEGs (**Figure 3**). Module sizes ranged from 12,585 genes in M1 to 343 genes in M11. Four hundred and ninety-seven genes were not clustered into any module, indicating that they did not share any common expression pattern with other co-expression modules. Out of the 11 co-expression modules, M1, M4, M7, M8, and M9 contain genes induced by virus infection, especially at the earliest sampling time point at 9 dpi (**Figure 3**). M1 contained genes strongly induced by viral infection at 9 dpi and then decreasing at later sampling times, while still elevated relative to the mock-inoculated plants. M4 contained genes that were initially highly expressed in virus-infected plants, with the highest levels observed in plants inoculated with WSMV. There was an increase of the M4 module genes in the control plants at 21 dpi. Genes present in M7 were induced by the double infections with a moderate upregulation of M7 genes at 21 dpi in WSMV-infected plants, whereas the expression levels of the M7 genes did not respond to mock and TriMV treatments (**Figure 3**). Genes categorized into M8 had the highest initial expression at 12 dpi in WSMV-infected plants and decreased in expression to be similar to the other viral treatments at 21 dpi, suggesting that the expression of the M8 genes was responding more exclusively to WSMV infections. M9 contained genes that displayed elevated expression in TriMV-infected plants, especially at 12 dpi, compared to WSMV and doubly infected plants.

In contrast, M2, M3, and M6 contained genes with reduced expression as a result of viral infection, with the level of suppression of gene expression corresponding to the phenotypic severity of symptoms observed, namely, TriMV < WSMV < Dual infection. Several modules also highlighted temporal impacts on gene expression. Modules M1, M6, and M10 showed elevated expression at 9 dpi, which gradually decreased over time in all four treatments. However, modules M3 and M5 contained genes that showed increased expression at 21 dpi in all four treatments (**Figure 3**). Overall, WGCNA analysis indicated gene expression responses broadly or specifically aligned with viral infection. To track these features, a domain and KEGG pathway enrichment analysis of all modules was performed, with a particular interest in modules M7, M8, and M9, which contained treatment-specific induced genes. A total of 93, 82, and 69 Interpro domains and 21, 11, and 2 KEGG pathways were enriched in M7, M8, and M9, respectively (**Supplementary Data Sheet 1**). M9 was enriched for genes associated with protein processing in the endoplasmic reticulum and plant-pathogen interaction pathways and genes encoding disease-resistant proteins like leucine-rich repeats (NBS-LRR), wall-associated receptor kinase, serine-threonine or protein kinase, NB ARC, heat shock proteins, and NAC transcriptional factor domains. While M8 was enriched with genes associated with glutathione metabolism, phenylpropanoid biosynthesis, and ABC transporter pathways and had significant enrichment in the transporter domain, enzymes like glutathione S-transferase, lysozyme-like domain, WRKY domain, and cellulose synthase. On the other hand, M7 was enriched with genes found in the lysosome; fatty acid degradation; MAPK signaling pathway – plant; and cutin, suberin and wax biosynthesis pathways and contained genes that were enriched proteinase inhibitor I12 Bowman-Birk domain, glycoside hydrolase, cysteine proteases-like cathepsin propeptide inhibitor domain, MADS-box transcription factor, leucine-rich repeats, and cysteine peptidase. Many of the proteins containing these domains play a major role in plant defense and anti-microbial activity.

### 3.4 Differentially expressed genes in wheat plants infected by WSMV, TriMV, or both viruses

**Figure 4:**
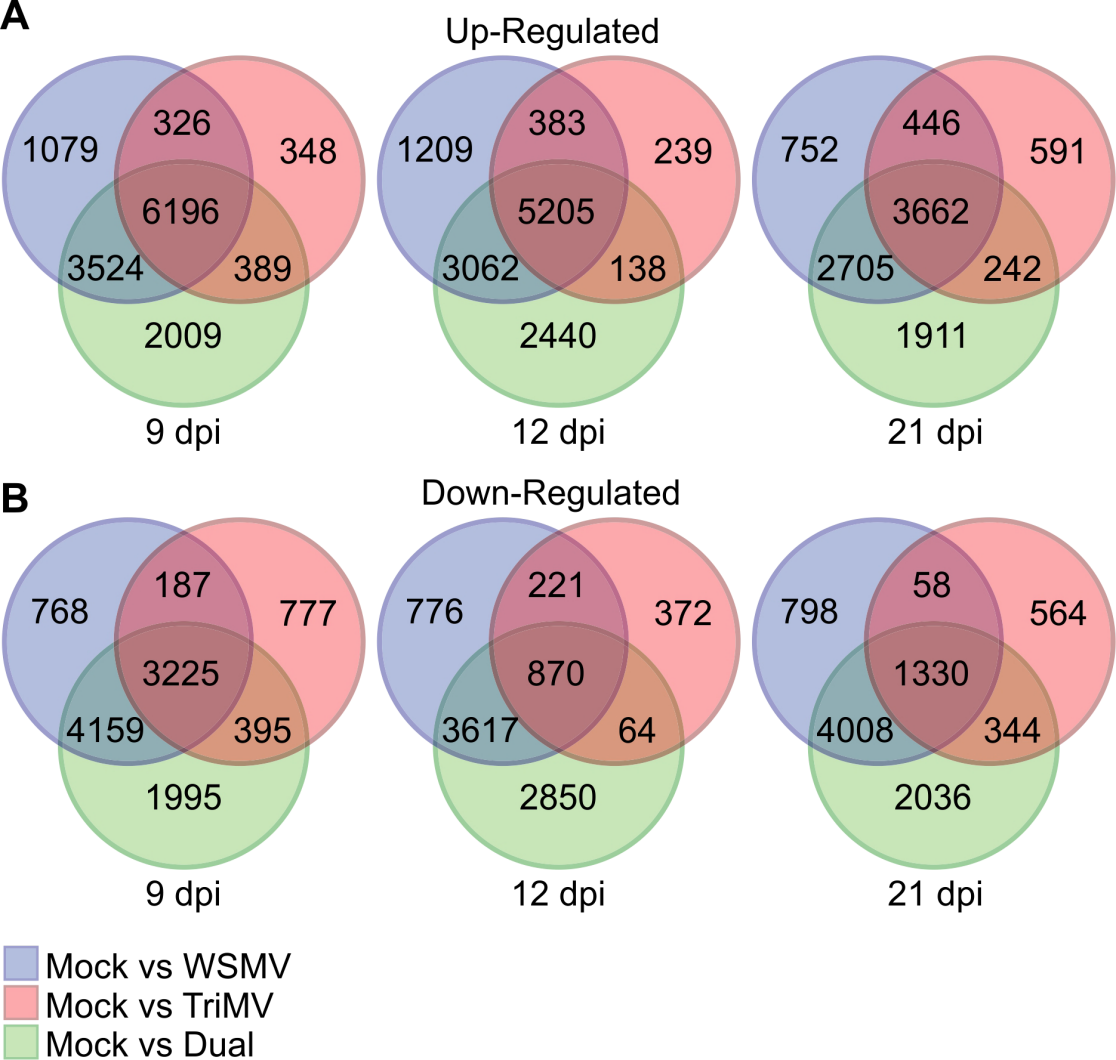
Venn diagrams of up- and down-regulated wheat genes in response to wheat streak mosaic virus (WSMV), Triticum mosaic virus (TriMV), or dual infection at 9, 12, and 21 days postinoculation (dpi). Venn diagrams representing **(A)** up-regulated and **(B)** down-regulated genes in wheat infected by WSMV, TriMV, or dual viruses at 9, 12, and 21 dpi.

Venn diagrams were used to visualize DEGs that were common and unique among single and double virus infections at each time point as compared to controls (**Figure 4**). Up-regulated DEGs shared between all three virus treatments decreased with the sampling date, starting with 6196 genes at 9 dpi to 3662 genes at 21 dpi (**Figure 4A**). Down-regulated DEGs shared between all three virus treatments were also greatest at 9 dpi (3225 genes) and decreased to 1330 genes at 21 dpi (**Figure 4B**). There were approximately 2000 DEGs unique per time point to the double infection across all the sampling dates. Approximately 3000 DEGs were shared in common between plants that were co-infected by both viruses and WSMV single infection at each time point, whereas <400 DEGs were shared in common between TriMV and WSMV or dual infections in pairwise comparisons (**Figure 4A**). These data suggest that the global transcriptomic changes in doubly inoculated wheat plants were more influenced by WSMV relative to TriMV. A similar scenario was observed for the down-regulated DEGs (**Figure 4B**).

Interpro domain and KEGG pathway enrichment analyses were used to glean functional information from the common gene sets (shared among all three treatments at the same time point) derived from the Venn diagrams. Significant enrichment was detected for 155, 221, and 157 Interpro domains and 8, 6, and 6 KEGG pathways in 9, 12, and 21 dpi virus-induced common gene sets, respectively (**Supplementary Data Sheet 1**). Significant enrichment in genes commonly induced by infection at all three time points was found in the protein processing in the endoplasmic reticulum and plant-pathogen interaction pathways. Further enrichment was detected in genes encoding cysteine-rich transmembrane domains, WRKY transcription factor domains, and NAC domains. Stress-responsive protein domains like late embryogenesis abundant protein, ribosomal protein L30e-like, and heat shock proteins (HSP) significantly increased their expression compared to healthy ones. Transcription gene regulators like VQ motifs play a vital role in the developmental process, signaling pathways, and biotic and abiotic stress. There were fewer domains enriched in commonly down-regulated genes, with 103, 51, and 70 Interpro domains and 17, 4, and 10 KEGG pathways enriched at 9, 12, and 21 dpi, respectively (**Supplementary Data Sheet 1**). The enriched KEGG pathways included photosynthesis, carotenoid biosynthesis, exopolysaccharide biosynthesis, and nitrogen metabolism. Other Interpro enriched domains included phytohormone-related domains like auxin/indole acetic acid domain and auxin response factor domain; transcription factors like MADS-box, Myc type, basic helix-loop-helix (BHLH), B3, and Myb; and photosynthesis-related genes like chlorophyll a/b binding protein and ferrodoxin 2Fe-2S family. Myc transcription factor domain-containing proteins play a crucial role in triggering jasmonic acid (JA) signaling in response to pathogen-associated stress (Ruan et al., 2019; Hewedy et al., 2023).

### 3.5 Gene ontology and KEGG analysis of commonly expressed DEGs in WSMV, TriMV, and doubly infected plants

**Figure 5:**
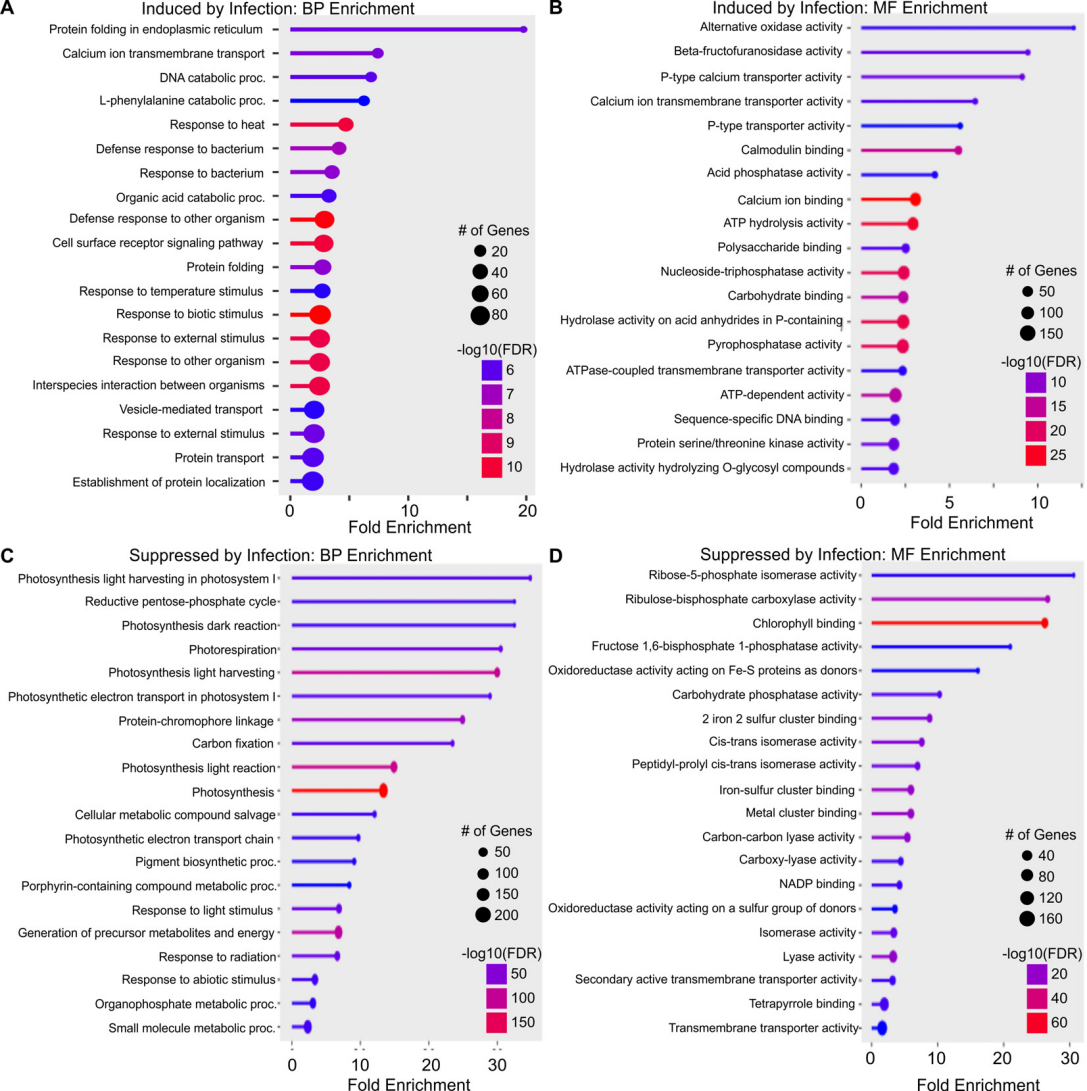
Gene ontology analysis of commonly expressed differentially expressed genes (DEGs) in wheat infected by wheat streak mosaic virus (WSMV), Triticum mosaic virus (TriMV), or dual viruses (WSMV+TriMV) at all time points. **(A)** Biological process (BP) enrichment of genes induced by infection; **(B)** Molecular function (MF) enrichment of genes induced by infection; **(C)** Biological process enrichment of genes suppressed by infection; and **(D)** Molecular function enrichment of genes suppressed by infection in all the treatments at all time points. The size of the dot represents the number of genes, and the color represents the enrichment.

Differentially expressed genes common in at least two treatments were considered for gene ontology enrichment analysis. KEGG pathway enrichment analysis of up-regulated genes detected 7 enriched pathways, including ribosome, phenylpropanoid biosynthesis, and plant-pathogen interaction (**Supplementary Data Sheet 1**). Gene ontology biological process enrichment of up-regulated DEGs disclosed that protein folding in the endoplasmic reticulum (GO:0034975) was significantly enriched with ~19-fold enrichment (**Figure 5A**). This class of proteins plays a crucial role in protein folding, which is required for cellular growth, development, and stress resistance. Calcium ion transmembrane transport (GO:0070588) and vesicle-mediated transport (GO:0070588) were also significantly enriched by infection (**Figure 5B**). Also enriched were DEGs involved in response to biotic stimulus (GO:0009607), biological processes involved in interspecies interaction between organisms (GO:0044419), response to external biotic stimulus (GO:0043207), and defense response to other organisms (GO:0098542), indicating broad plant defensive responses to viral infection. Many genes associated with the transport process and localization were enriched (**Figure 5A**).

The molecular function gene ontology of upregulated genes is shown in **Figure 5B**. The greatest fold enrichment was seen for alternative oxidase activity (GO:0009916), beta-fructofuranosidase activity (GO:0004564), and P-type calcium transporter activity (GO:0005388). Molecular function gene ontology showed greater numbers of genes associated with functions, such as calmodulin binding (GO:0005516), acid phosphatase activity (GO:0003993), and ATP-dependent activity (GO:0140657). Genes associated with protein serine/threonine kinase activity (GO:0004674) were enriched (**Figure 5B**).

KEGG pathway enrichment analysis of down-regulated genes detected 17 enrichment pathways, including photosynthesis, diterpenoid biosynthesis, plant hormone signal transduction, nitrogen metabolism, and carbon metabolism (**Supplementary Data Sheet 1**). The biological processes enriched in DEGs suppressed by infection were mostly involved in photosynthesis pathways like photosynthesis light harvesting in photosystem I (GO:0009768), photosynthesis light harvesting (GO:0009765), and photosynthesis dark reaction (GO:0019685). Also, DEGs in pathways reductive pentose-phosphate cycle (GO:0019253) and carbon fixation (GO:0015977) had greatly reduced expression (**Figure 5C**). These data suggest that carbon fixation and assimilation were much lower in doubly infected plants than in control plants, consistent with the phenotypic observations over all three sampling time points.

Molecular function enrichment analysis highlighted many genes associated with carbon assimilation and those needed for iron-sulfur cofactor biosynthesis such as ribose-5-phosphate isomerase activity (GO:0004751), oxidoreductase activity acting on Fe-S proteins as donors (GO:0016667), chlorophyll-binding (GO:0016168), 2-iron 2-sulfur cluster binding (GO:0051537), and fructose 1,6-bisphosphate 1-phosphatase activity (GO:0042132) were significantly down-regulated as well (**Figure 5D**).

### 3.6 Gene ontology and KEGG analysis of DEGs unique to double infections

**Figure 6:**
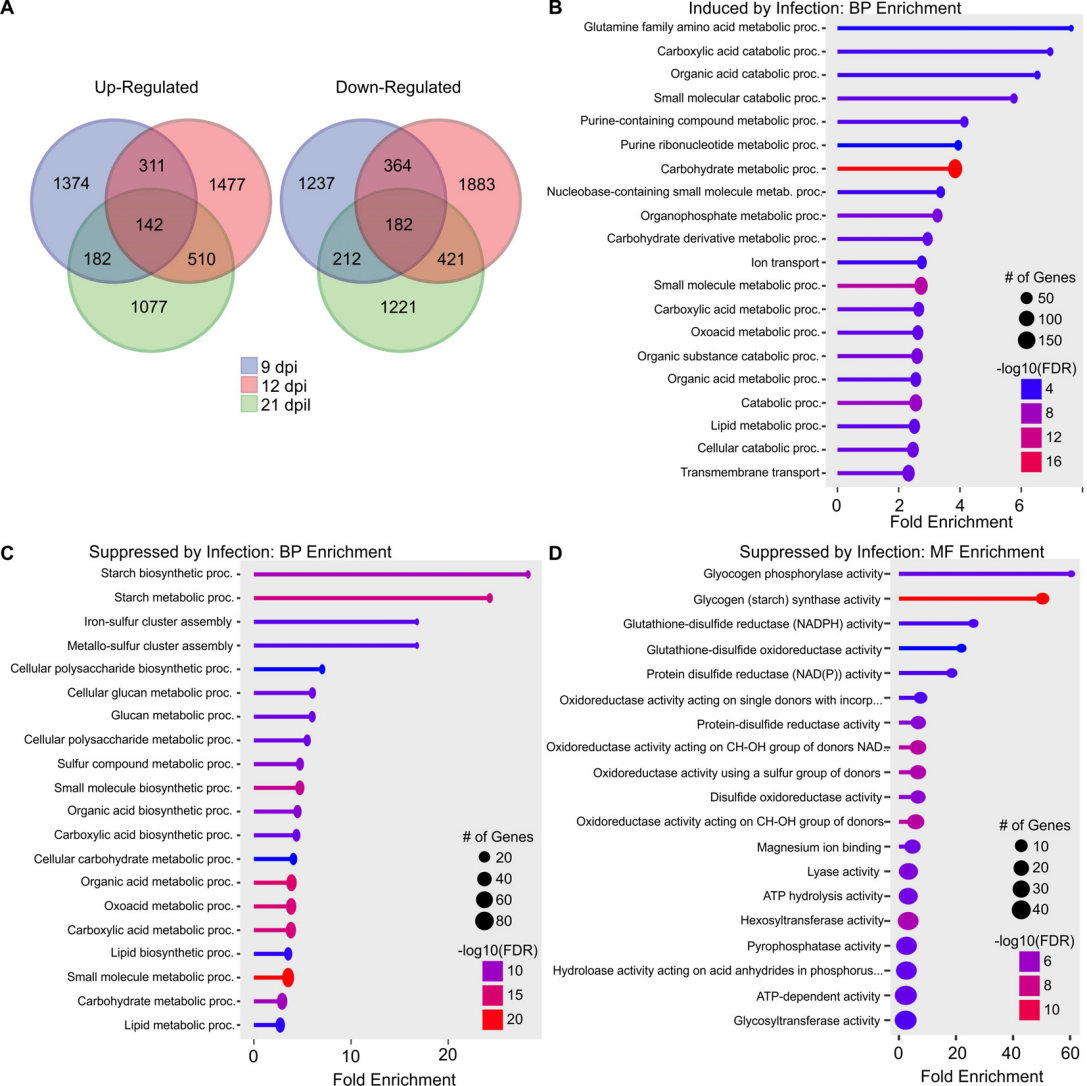
Gene ontology analysis of differentially expressed genes (DEGs) unique to co-infected wheat by wheat streak mosaic virus and Triticum mosaic virus at two or more time points. **(A)** Venn diagrams representing the uniquely expressed genes in wheat infected by dual viruses at 9 days postinoculation (dpi), 12 dpi, and 21 dpi. Biological process (BP) enrichment of genes induced **(B)** and suppressed **(C)** by infection only in dual virus infection at two or more time points; **(D)** Molecular function (MF) enrichment of genes suppressed by only in dual virus infection at two or more time points. The size of the dot represents the number of genes, and the color represents the enrichment.

Differentially expressed genes detected only in double infections were of particular interest because they could contain wheat genes that might underlie the severe phenotypic responses to infection. Plants infected with both WSMV and TriMV had 2009, 2440, and 1911 DEGs uniquely induced by infection at 9, 12, and 21 dpi, respectively, with 142 DEGs common in all three time points (**Figures 4**, **6A**). In dual-infected wheat, 1995, 2850, and 2036 DEGs were uniquely suppressed by infection at 9, 12, and 21 dpi, respectively, with 182 DEGs in common for all three time points (**Figures 4**, **6A**). Genes that were common with at least two sampling time points were used for enrichment analysis. KEGG pathway enrichment analysis of up-regulated DEGs detected 5 enriched pathways, including N-Glycan biosynthesis; valine, leucine and isoleucine degradation; and alanine, aspartate and glutamate metabolism (**Supplementary Data Sheet 1**). Biological process gene ontology enrichment analysis of up-regulated DEGs induced by double infection revealed that several catabolic processes were upregulated (**Figure 6B**). For example, the glutamine family amino acid metabolic process (GO:0009064) was significantly enriched with 7.63-fold enrichment. This family of amino acids plays an important role in signal transduction and nitrogen assimilation. In addition, the purine-containing compound metabolic process (GO:0072521) and carboxylic acid metabolic process (GO:0019752) had the highest number of genes that were induced by dual-virus infection (**Figure 6B**). No significant enrichment was found in the molecular function of up-regulated genes.

KEGG pathway analysis of down-regulated DEGs detected 4 enriched pathways, including starch and sucrose metabolism and, flavone and flavonol biosynthesis (**Supplementary Data Sheet 1**). Gene ontology analyses of down-regulated DEGs indicated that the starch biosynthetic process (GO:0019252) and iron-sulfur cluster assembly (GO:0016226) were negatively regulated in response to double virus infection (**Figure 6C**). Molecular function gene ontology enrichment analysis revealed that glutathione-disulfide reductase (NADPH) activity (GO:0004362) and glutathione-disulfide oxidoreductase activity (GO:0015038) were also negatively regulated (**Figure 6D**). Glutathione reductases play a crucial role in the control of reactive oxygen species (ROS). It was evident that the presence of two viruses influenced a wide swath of genes required for normal growth processes as compared to single virus infections. Damage from TriMV infection was relatively less severe phenotypically and transcriptionally; its combination with WSMV in dual virus infections led to greater negative changes to the wheat transcriptome, leading to greater disease severity than WSMV alone.

### 3.7 Transcription factors play a key role in disease synergism in co-infected wheat plants

**Figure 7:**
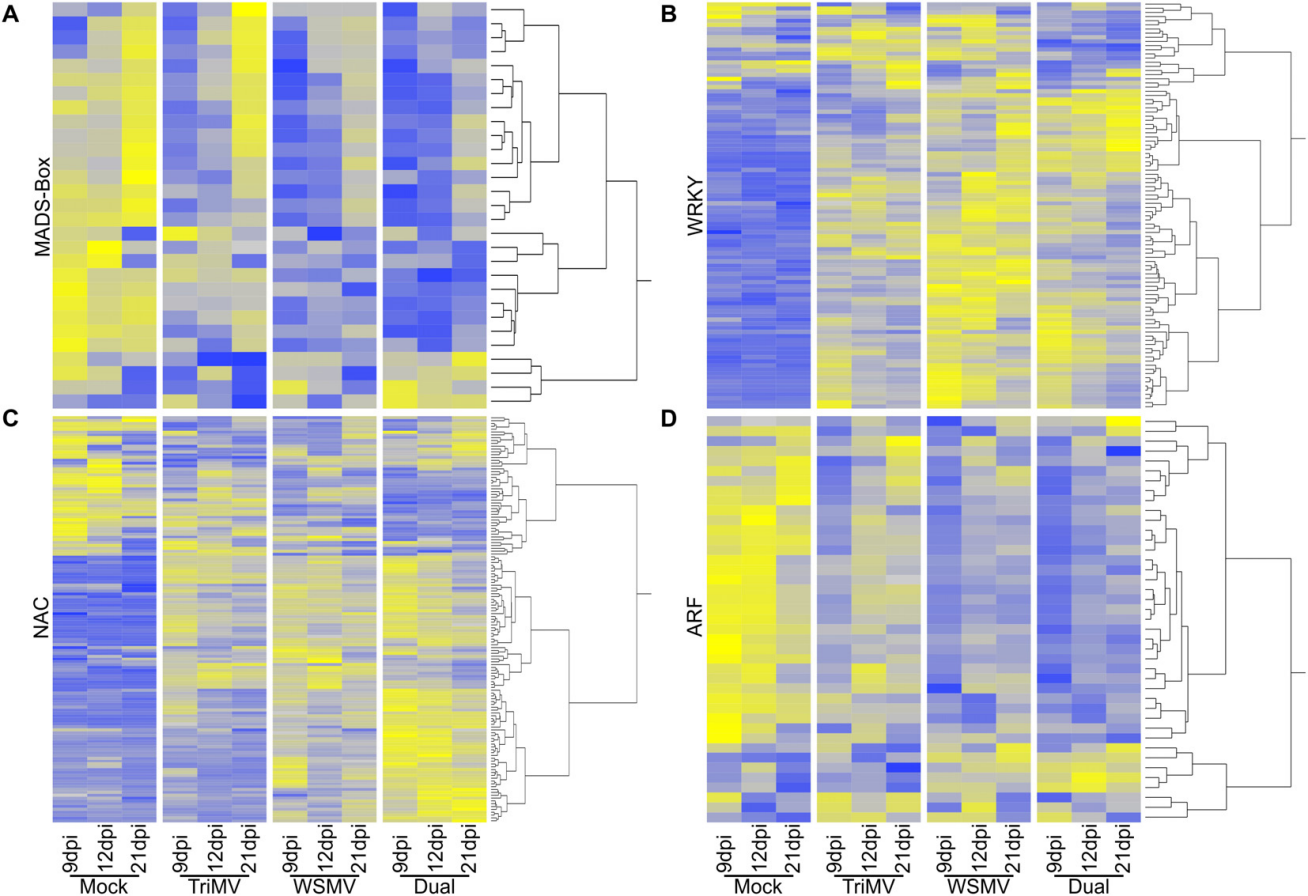
Heat maps representing transcription factor expression in wheat infected by wheat streak mosaic virus (WSMV), Triticum mosaic virus (TriMV), or dual viruses (WSMV+TriMV) at all time points. The responses of the genes were mapped from blue to white to yellow with a z-score of -2 to 0 to +2, where blue represents low expression, grey represents intermediate expression, and yellow represents high expression. **(A)** MADS-box transcription factors; **(B)** WRKY transcription factors; **(C)** NAC transcription factors; and **(D)** ARF transcription factors.

Transcription factors (TFs) play a crucial role in regulating the expression of genes related to plant growth and stress. Plants contain several families of TFs, and members of many families, such as MADS-Box, ARFs, and NACs can be associated with multiple plant developmental events (Mitsuda and Ohme-Takagi, 2009). In contrast, WRKYs have been implicated to have a determining role in plant biotic stress responses (Saha et al., 2023). DEGs representing four major families of TFs were analyzed to determine whether changes in TF expression might be tracked with single or double infections (**Figure 7**).

MADS-Box genes are usually associated with growth, flowering, and fruiting in plants (Adhikari and Kasahara, 2024). A total of 29 MADS-Box genes were differentially expressed in our dataset (**Figure 7A**). Of these 29 genes, 25 were highly expressed in control plants at all sampling time points. One gene was downregulated at 12 dpi in control plants relative to its expression levels at 9 dpi. Only two MADS-Box genes were highly expressed by TriMV infection at 9 dpi (**Figure 7A**). While there were limited changes at 12 dpi in MADS-Box gene expression, 22 genes were found with higher abundances at 21 dpi in TriMV-infected plants; notably, transcripts for many of these genes were also greater at the two later sampling times in control plants (**Figure 7A**). WSMV infection caused a MADS-Box gene expression profile quite similar to plants singly infected with TriMV, with four, six, and 16 MADS-Box genes with higher expression at 9, 12, and 21 dpi, respectively (**Figure 7A**). For dual infections, these numbers were five, four, and 12 MADs-Box genes with higher expression at 9,12, and 21 dpi, respectively (**Figure 7A**). Notably, two MADS-Box genes with the highest transcript abundances at 9 dpi in dual-infected plants were moderately induced by TriMV and WSMV in single infections.

A total of 97 WRKYs were differentially expressed among the sampling time points and treatments (**Figure 7B**). WRKYs were mostly downregulated in mock-inoculated plants (controls), although a few WRKYs were upregulated in controls on different sampling dates (**Figure 7B**; **Supplementary Data Sheet 1**). TriMV infection induced 48 WRKY at 9 dpi, of which expression of 47 WRKYs remained elevated at 12 dpi, and several of these WRKYs had peak expression at 12 dpi. Notably, 12 WRKYs had the greatest expression at 21 dpi in TriMV-infected plants, and many of the other WRKY genes upregulated at earlier sampling time points were expressed at lower levels (**Figure 7B**). Infection by WSMV induced the expression of 76 WRKYs at 9 dpi; several of these genes had higher expression levels in WSMV-infected plants compared to TriMV-infected plants. Induction of 16 new WRKY encoding genes was observed in plants infected with WSMV at 12 dpi. Transcript levels of most of the WRKY-encoding genes induced at earlier sampling time points remained high at 21 dpi in WSMV-infected plants (**Figure 7B**). Dual infection of wheat with TriMV and WSMV resulted in the induction of 64 WRKY encoding genes at 9 dpi, with a majority of genes common to single infections by WSMV. However, 11 WRKY encoding genes had higher expression at 9 dpi in dual-infected plants relative to single infections. Transcript levels for most WRKY genes remained elevated at all sampling time points in dual infection (**Figure 7B**).

For wheat genes encoding differentially expressed NAC TFs, transcripts for 48 out of 143 genes were more abundant at 9 dpi in controls, and conversely, 95 NAC encoding genes were downregulated (**Figure 7C**). At 12 dpi, 12 NAC genes were expressed at higher levels compared to their transcript abundances at 9 dpi, while the other NAC genes had generally similar or somewhat lower transcript abundances, a trend that continued at 21 dpi in control plants (**Figure 7C**). Single and dual virus infections significantly changed the numbers and differential expression of NAC genes (**Figure 7C**). In total, 80 NACs were induced at 9 dpi in TriMV infection, with the numbers decreasing to 58 and 44 at 12 and 21 dpi, respectively. Among these, only 14 NACs had higher levels of expression at 12 dpi. Transcripts for only 6 NACs were more abundant at 21 dpi in TriMV-infected plants relative to earlier time points. WSMV infection induced the expression of 89 NAC encoding genes at 9 dpi, a number like those seen for TriMV-infected plants, although most of the genes with the greatest transcript counts were largely unique to WSMV infections (**Figure 7C**). Transcripts for most of these genes were less abundant at the next two sampling time points, suggesting a response unique to WSMV. However, transcripts for 24 and nine other NAC encoding genes were more abundant at 12 and 21 dpi, respectively, in WSMV-infected wheat. Dual virus infection caused strong increases in transcript abundances for 77 NAC encoding genes at 9 dpi (**Figure 7C**), many of which were upregulated by WSMV infection as well, although there appeared to be a synergistic interaction between the two viruses. Several of these genes remained highly expressed at 12 and 21 dpi, providing some evidence for the underlying causes of disease synergism observed in the dual infection.

The number of DEGs for ARF and MADS-box encoding genes was less than those seen for WRKYs and NACs. For ARF TFs, there were a total of 41 DEGs, of which a total of 34 were induced and the rest were repressed at 9 dpi in control plants (**Figure 7D**). Most of these ARF-encoding genes remained more highly expressed at 12 dpi (33 genes) and 21 dpi (26 genes) in control plants. Nine ARFs had higher transcript abundances at 9 dpi in TriMV-infected plants, of which only two were repressed in controls (**Figure 7D**). Notably, 28 ARFs were enriched at 12 dpi in TriMV-infected plants, of which four were repressed at 12 dpi in controls. In a distinct contrast, WSMV infection induced six ARF genes at 9 dpi, of which three genes were upregulated uniquely upon WSMV infection. Similar to data from TriMV-infected plants, transcripts for 15 ARFs were higher at 12 dpi in WSMV-infected plants (**Figure 7D**). Dual infections also induced six ARF genes, of which four were common only to WSMV infection. At 12 and 21 dpi, ARF genes observed with higher transcript abundances at 9 dpi were unchanged, except for one gene, which was upregulated at 21 dpi in dual infection.

## 4 Discussion

Synergistic interactions between unrelated viruses have been documented in several plant-virus combinations (Syller, 2012; Redinbaugh and Stewart, 2018; Moreno and López-Moya, 2020). Frequently, these synergistic interactions result in an increase in symptom phenotype and improvement in several viral parameters such as replication rate, movement within host cells, cytopathological effects, transmission, and titers (Mascia and Gallitelli, 2016). Wheat curl mites transmit both WSMV and TriMV and can readily co-infect field-grown wheat (Byamukama et al., 2013; Albrecht et al., 2022). Mixed infections under field settings can be expected and can cause significant yield losses (Moreno and López-Moya, 2020). The discovery of plant genes and pathways that are significantly impacted by single or double viral infections could provide new information for breeding more resilient wheat varieties.

Several studies have identified transcriptomic changes induced by viral infections (Kumar et al., 2021; Sharaf et al., 2023; Xu et al., 2024). In *Cucumis sativus* infected with cucumber green mottle mosaic virus, there was an early downregulation of WRKYs at 3 dpi, followed by a strong upregulation in WRKYs at 21 dpi. NACs were both up- and downregulated at both sampling time points. In rice tungro bacilliform virus-infected rice plants, a range of host responses centered on defense, such as PR proteins, those associated with cell walls, and secondary metabolites, were induced. Among TFs, WRKYs, MYB, and ERFs were induced. In both virus-infected cucumber and rice, plant genes required for SA, JA, and ethylene biosynthesis were differentially induced (Kumar and Dasgupta, 2020; Slavokhotova et al., 2021). In *Brachypodium*, infection with the synergistic viruses, panicum mosaic virus and its satellite virus caused the upregulation of genes associated with carbon metabolism, responses to stress, and metabolite transport, among others, and downregulated genes associated with photosynthesis (Mandadi and Scholthof, 2012). There also was a strong upregulation of genes encoding chaperones/heat shock proteins (HSPs), proteases, and cytochrome p450s. Significantly, several WRKYs were induced by the dual virus infections (Mandadi and Scholthof, 2012). These data point to significant similarities in monocots (rice, *Brachypodium*) and dicots (*Cucumis*) responses to viral infection.

This study substantially reinforces many of the commonalities of plant defense responses to viral infection and yet provides important clues on changes in global gene expression that are unique to single or dual virus infections in wheat. For example, WGCNA analyses determined that a cohort of DEGs responding specifically to single and dual infections and co-expression modules containing DEGs upregulated by dual infections were enriched in genes associated with plant defense and disease signaling. Such genes included cysteine- and leucine-rich transmembrane receptors/receptor kinases (NB-LRRs) (Kuramata et al., 2009; Venancio and Aravind, 2010; Pereira Mendes et al., 2021), heat shock proteins (Kumar and Dasgupta, 2020), and glutathione reductases, among others. Plant phenotypic observations showed mild, moderate, or severe negative effects on plant growth and tiller production in plants infected singly or doubly with TriMV and WSMV. The double infections significantly retarded plant development and enhanced senescence, consistent with lowered expression of genes associated with plastid function and upregulation of genes associated with plant senescence. Analysis of 4 classes of TFs indicated that MADS-Box encoding genes had higher expression in control plants at all sampling time points. MADS-Box genes are associated with a number of growth and developmental events (Adhikari and Kasahara, 2024). As an example, one of the most highly expressed MADS-Box encoding genes at 9 dpi in control plants, TraesCS3D02G427700, is orthologous to AT4G11880 (AGL14) which has been implicated in auxin transport and shoot apical meristem transitions (Garay-Arroyo et al., 2013; Perez-Ruiz et al., 2015). TraesCS3D02G427700, is significantly downregulated at 21 dpi in WSMV-infected plants and at 9 dpi in doubly infected plants, consistent with suppression in plant growth. It has been reported that a MADS-box gene provided resistance to soybean mosaic virus in soybean plants (Ren et al., 2022). However, both phenotypic and transcriptomic data do not currently support a similar role for a MADS-Box gene, at least in our system.

Two other major classes of TFs, namely NACs and ARFs, were differentially expressed in control and infected plants. An ARF, encoded by TraesCS7B02G363100, was significantly upregulated in control plants at all sampling times and conversely downregulated in WSMV and doubly infected plants. This ARF is an ortholog of AT4G30080, which impacts auxin and JA signaling in relation to ABA during seed germination in *Arabidopsis* (Bascom, 2023). Transcriptomic data supported changes in the expression of genes associated with hormone biosynthesis and signaling as a result of viral infection of wheat plants. Downregulation of auxin and JA signaling pathways in plants can increase disease susceptibility. These hormones are vital for the plant’s defense mechanisms, and their suppression allows pathogens to invade more easily (Collum and Culver, 2016). In rice, increased susceptibility due to reduced auxin signaling was linked to a decrease in JA signaling, indicating that auxin signaling may protect against viruses by activating JA-dependent defenses (Zhang et al., 2019).

A total of 142 NACs were part of the DEGs, of which 48 and 92 had increased expression at 9 dpi in control and doubly infected plants, respectively. As a few examples, the NAC, encoded by TraesCS1B02G272600, is an ortholog of the *Arabidopsis* NAC AtLOV1, which indirectly controls vegetative growth by influencing flowering time (Yoo et al., 2007). The observation that the wheat ortholog had higher expression in control and TriMV-infected plants (both had good growth) compared to WSMV and dually infected plants (with suppressed growth) would suggest a role for this wheat NAC in promoting vegetative growth. Conversely, over 20 NACs had higher expression in dual infections at 9 dpi relative to control and singly infected plants. These included orthologs to different *Arabidopsis* NACs variously implicated in promoting leaf senescence, cell death, and growth repression, consistent with changes observed in plant growth.

Our observations from this study show that WRKY transcription factors were mostly upregulated in all virus treatments except in TriMV-infected plants. Out of a total of 98 differentially expressed WRKYs, only 12 were upregulated in control plants at 9 dpi, with many downregulated at other sampling times. Although WRKYs can have roles in normal plant development, they have been most often discovered to be important regulators of plant defense, with transcriptional activating or transcription repressing properties (Saha et al., 2023; Gao et al., 2024; Guo et al., 2024). In this study, among the wheat WRKY genes upregulated at 9 dpi in doubly infected plants were orthologs to *Arabidopsis* WRKY55 (AT2G40740; TraesCS4A02G193600), WRKY 65 (AT1G29280; TraesCS3A02G281900), and WRKY33 (AT2G38470; TraesCS1D02G072900). ATWRKY55 accelerates leaf senescence and influences plant defense through transcriptional control of genes encoding proteins required for ROS and SA (Wang et al., 2020). It is plausible that the deleterious effects on plant growth by dual infection could have been partially due to the wheat WRKY55 ortholog. Notably, expression levels of TraesCS4A02G193600 were elevated at all three sampling dates in plants doubly infected with WSMV and TriMV, only elevated at 9 dpi in WSMV-infected plants and reduced in control and TriMV-infected plants, lending some support for its role in disease synergism. ATWRKY33 and its orthologs have roles in several aspects of plant metabolism, including plant defense (Chen and Zhang, 2024; Tao et al., 2024), plausibly TraesCS1D02G072900 appears to play a similar role in viral infection in wheat plants. Overall, our data suggested a significant role for specific TFs, principally NACs and WRKYs, in disease progression, especially in plants with dual virus infection.

To more closely examine the WSMV-TriMV synergism, genes with significantly differential expression between dual infection and both single infections, but not single infections and mock, were analyzed (**Supplementary Data Sheet 1**). This analysis indicated that 374 genes were differentially upregulated in at least one of the three time points, but only 17 genes were consistently upregulated in plants doubly infected with WSMV and TriMV at all three sampling times. Among these 17 genes was a wheat ortholog to *Arabidopsis* NAC083, which interacts with mungbean yellow mosaic India virus, potentially improving virus replication (Suyal et al., 2014), a homeobox ATHB12 ortholog that improves geminivirus infection (Park et al., 2011), a RelA/SpoT ortholog that is required for proper chloroplast functions and can interact with TOR to regulate growth (D'alessandro et al., 2024), a transcription initiator, and a gene encoding a 1-phosphatidylinositol-3-phosphate 5-kinase of unknown function. Notably, a gene encoding a glucan endo-1,3-beta-glucosidase 1, that in *Arabidopsis* (AT1G69295) can bind to callose and influence cell-to-cell trafficking (Simpson et al., 2009), and an Arabidopsis pectate lyase (PMR6; AT3G54920) ortholog which is required for susceptibility to powdery mildew (Vogel et al., 2002) were also found. The fact that many of these orthologous genes have been associated with plant defense or viral replication is encouraging with the potential to more directly associate their roles in wheat to viral susceptibility and disease synergism.

The 16 genes that were specifically downregulated at all sampling time points in doubly infected plants included a peroxidase, a WRKY 40 ortholog, a cytochrome P450, starch synthases, and an elicitor-induced cinnamyl alcohol dehydrogenase (**Supplementary Data Sheet 1**). WRKY40 (AT1G80840) is a transcriptional repressor associated with ABA and chloroplast functions in *Arabidopsis* (Liu et al., 2013), and the cytochrome P450 (AT5G36110) encodes an enzyme that oxidizes triterpenes and lowers their defensive potential (Yasumoto et al., 2016). Conceivably, changes in chloroplast functions and lowered plant growth are associated with the downregulation of these wheat genes. The upregulated and downregulated genes may serve as valuable targets to investigate interactions between viral and host factors, enhancing our understanding of viral synergism in wheat.

**Figure 8:**
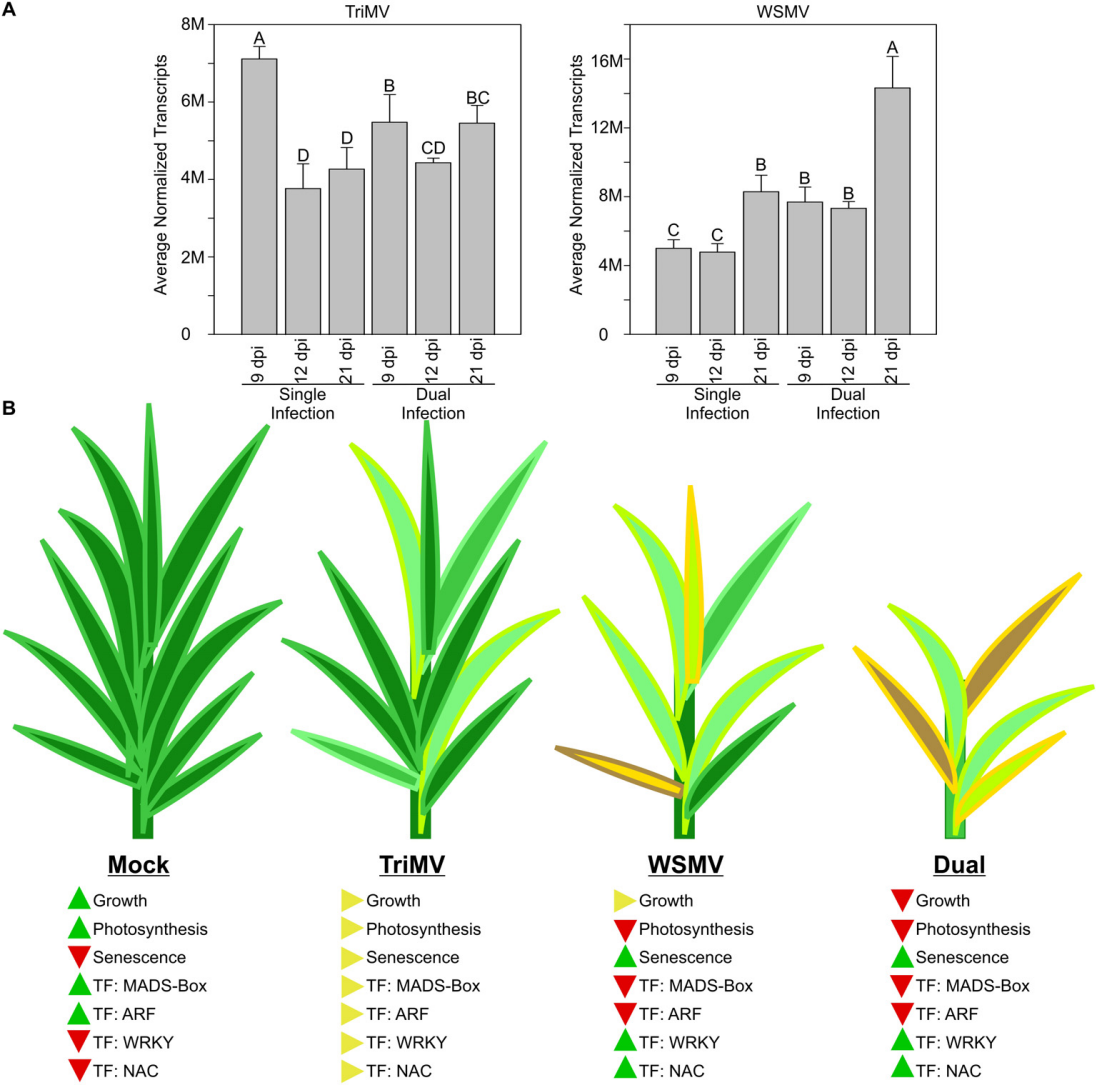
Summary of the number of viral transcripts and expression of wheat genes and transcription factors (TF) in Triticum mosaic virus (TriMV), wheat streak mosaic virus, or dual virus (WSMV+TriMV)-infected wheat. **(A)** The number of transcripts specific to TriMV and WSMV from single and dual infections in wheat at 9 days postinoculation (dpi), 12 dpi, and 21 dpi. **(B)** Cartoon diagrams of wheat plants of mock, TriMV, WSMV, and dual virus infection at 21 dpi. The levels of expressed genes related to growth, photosynthesis, and senescence, as well as TF of MADS-Box, ARF, WRKY, and NAC, are indicated with arrowheads. The levels of accumulation were indicated with upward (increase), side (no change), and downward (decrease) arrowheads.

In conclusion, synergistic interactions between WSMV and TriMV significantly impacted viral transcript levels (**Figure 8A**), drastically enhancing disease phenotype compared to individual virus infections. The damage to plants infected only with TriMV was minimal compared to those infected with WSMV. Plant outcomes, changes in development, and TFs are summarized in **Figure 8B**. As expected, in mock (control) plants, genes associated with growth and photosynthesis were upregulated, while those related to senescence were downregulated. Critically, most MADS-Box and ARF TFs were upregulated and many NACs and WRKYs were downregulated in mock-inoculated plants. In wheat leaves singly infected with TriMV, most of the major plant factors were moderately impacted. In plants singly infected with WSMV, genes associated with growth had intermediate expression, those encoding genes required for photosynthesis, MADS-Box, and ARFs were downregulated, and genes associated with senescence and encoding WRKYs and NACs were upregulated, indicating that WSMV infection had a greater impact on wheat transcriptomes relative to TriMV infection. However, the impact of viral synergism was quite evident in dual infections. Genes needed for growth, photosynthesis, MADS-Box, and ARFs were significantly downregulated, whereas those associated with senescence and those encoding NACs and WRKYs were significantly upregulated, indicating the strong disruption to plant metabolism and likely channeling of energy and metabolites for viral replication. There also appeared to be a connection between viral replication and plastid health, with stronger downregulation of genes needed for chloroplast functions and integrity and increased synergism between TriMV and WSMV. The underlying reasons for these findings in wheat are yet to be explored.

## Data Availability

The datasets presented in this study can be found in online repositories. The names of the repository/repositories and accession number(s) can be found in the article/**Supplementary Material**.
